# Relationship between oral frailty and locomotive syndrome in working-age individuals: a cross-sectional survey of workers in Japan

**DOI:** 10.1186/s12903-023-03453-6

**Published:** 2023-10-04

**Authors:** Rie Nagao-Nishiwaki, Akinobu Nishimura, Makoto Ohtsuki, Toshihiro Kato, Akihiro Sudo

**Affiliations:** 1 Department of Nursing, Faculty of Nursing, Suzuka University of Medical Science, 3500-3 Minamitamagaki-Cho, Suzuka City, Mie, 513-8670 Japan; 2 Department of Orthopaedic and Sports Medicine, Mie University Graduate School of Medicine, 2-174 Edobashi, Tsu City, Mie, 514-8507 Japan; 3 Department of Clinical Nutrition, Faculty of Health Science, Suzuka University of Medical Science, 1001-1 Kishioka-Cho, Suzuka City, Mie, 510-0293 Japan; 4 Department of Rehabilitation, Suzuka Kaisei Hospital, 112-1 Kou-Cho, Suzuka City, Mie, 513-8505 Japan; 5 Department of Orthopaedic Surgery, Mie University Graduate School of Medicine, 2-174 Edobashi, Tsu City, Mie, 514-8507 Japan

**Keywords:** Frailty, Oral health, Tooth loss, Mastication, Locomotive syndrome, Sarcopenia

## Abstract

**Background:**

Although the relationship between oral and physical frailty in older adults has been investigated, few studies have focused on the working-age population. This study examined the relationships of the number of remaining teeth and masticatory ability, i.e., signs of oral frailty, with locomotive syndrome (LS) in the working-age population.

**Methods:**

The number of remaining teeth, masticatory ability, and presence of LS in 501 participants from four companies were examined. The relationships between the number of remaining teeth groups (≥ 20 teeth or ≤ 19 teeth) and LS and between the masticatory ability groups (high or low) and LS were examined. A binomial logistic regression analysis was conducted using LS from the stand-up test as the objective variable and the two subgroups based on the number of remaining teeth and potential crossover factors as covariates.

**Results:**

The analysis included 495 participants (354 males and 141 females; median age, 43 years). The median number of remaining teeth among the participants was 28, and 10 participants (2.0%) had ≤ 19 teeth. The mean masticatory ability values were 39.9 for males and 37.7 for females, and 31 participants (6.3%) had low masticatory ability. In the stand-up test, those with ≤ 19 teeth had a higher LS rate than those with ≥ 20 teeth. The odds ratio for LS in the group with ≤ 19 remaining teeth was 5.99, and the confidence interval was 1.44–24.95.

**Conclusions:**

The results confirmed signs of oral frailty in the working-age population. Further, the number of remaining teeth possibly affects standing movement. Thus, oral frailty is associated with LS in the working-age population.

## Background

As the population ages, increasing healthy life expectancy is a goal in Japan. As oral health is important in maintaining overall health [1], age-related oral frailty and its prevention have received considerable attention. In 2013, oral frailty was defined by the Japanese Dental Association (JDA) as a set of phenomena and processes in which age-related changes in various oral conditions (number of teeth, oral hygiene, oral function, etc.), combined with decreased oral health awareness and physical and mental reserve capacity, lead to increased oral frailty and eating dysfunction, resulting in a decline in physical and mental function [2].

There is a lack of interest in oral health in the early stages of oral frailty, and the risk of tooth loss appears. In the advanced stages, a decline in the masticatory ability as well as the development of sarcopenia, a loss of muscle mass throughout the body, and locomotive syndrome (LS), a loss of mobility functions such as standing and walking due to disorders of the locomotor system (bones, joints and discs, muscles and nervous system), are believed to occur.

The concept of LS was proposed by the Japanese Orthopaedic Association (JOA) in 2007: it is caused by disorders of the locomotor system, with symptoms such as pain, limited joint range of motion, joint deformity, reduced balance ability, and slow walking speed, which increases with age [3, 4]. Musculoskeletal conditions, such as falls, fractures, and joint disease, account for approximately 20% of the care needs of older people, and approximately 67% of general people have LS [5]. LS stage 1, the early stage of LS, indicates that muscle strength and balance are beginning to decline, whereas LS stage 3 is almost similar to physical frailty, with a severe decline in mobility that progresses to a state wherein it interferes with social participation.

Factors contributing to LS include female sex, older age, low body weight, obesity, low physical activity, lack of regular exercise, low frequency of current smoking, and low frequency of current drinking habits. The biological relationship between physical frailty and oral frailty in elderly people has been extensively studied, and the evidence base for relevant oral indicators is growing. Physical frailty and oral frailty in older people are most frequently associated with the number of remaining teeth, followed by masticatory function [6]. Furthermore, falls have also been reported to be associated with the number of remaining teeth. Additionally, the relationship between LS, as assessed by a questionnaire, and oral frailty in the elderly has been reported to be affected by the number of remaining teeth, low masticatory ability, and the presence of subjective mastication and swallowing symptoms in patients with LS [7–10].

LS has traditionally been considered a problem for the elderly population, but it has recently been shown that a considerable number of people in the working-age population are affected by LS [11–14]. In the latest report on the prevalence of LS in the working-age population, it was reported that LS stage 1 was present in 21.7% and 25.0% of males and females younger than 40 years of age, 31.6% and 41.1% of those aged 40–49 years, and 41.5% and 42.1% of those aged 50–59 years, respectively [5].

LS has been identified in the working-age population, which means that signs of oral frailty, believed to occur before LS, can be detected, as physical frailty and oral frailty are associated, but few studies have investigated this. The hypothesis of this study was that the working-age population with LS would show signs of oral frailty. Therefore, this study aimed to determine the relationship between LS and the number of remaining teeth and masticatory ability, which are closely related to oral frailty in the working-age population.

## Methods

### Setting and participants

**Fig. 1:**
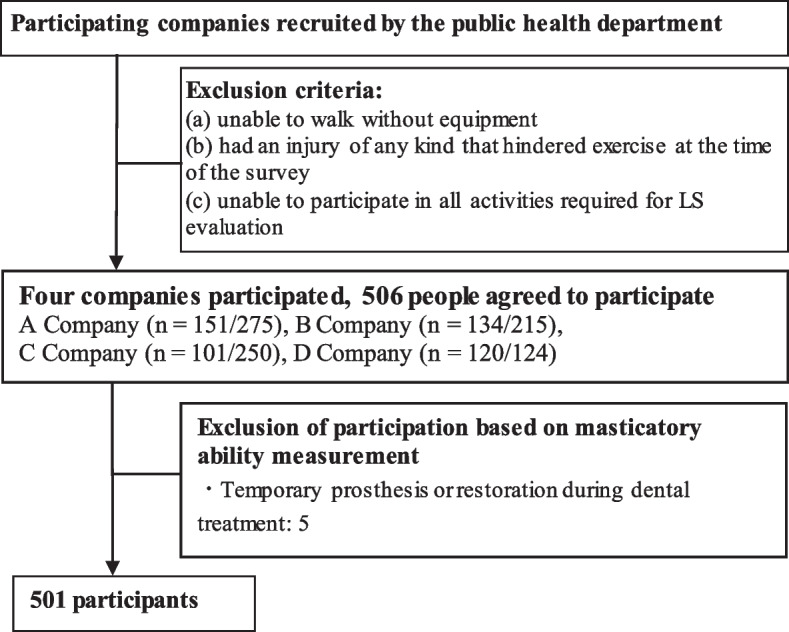
Flowchart of the participant selection process

A cross-sectional study was performed at four manufacturing companies recruited by the public health department of a local prefecture in Japan that agreed to participate from 2015 to 2019 (Fig. 1). The companies included were a chemical company (company A, 275 day-shift workers), an electronics company (company B, 215 day-shift workers), and two pharmaceutical companies (company C, 250 day-shift workers; company D, 124 day-shift workers). Each of the following periods were surveyed: for company A, 2 days in 2018; for company B, 2 days in 2019; for company C, 2 days in 2018; and for company D, 1 day in 2017. Measurements were taken in a room in each company. The JOA-prescribed method of assessing LS was used, and to control for informational bias, a trained investigator performed the assessment. Participants were not eligible to take part in the survey if they (a) were unable to walk without equipment (e.g., T-canes, crutches, wheelchairs), (b) had an injury of any kind that hindered exercise at the time of the survey, and (c) were unable to participate in all activities for LS evaluation. Each company informed its employees before their eligibility was assessed and only recruited volunteers. Overall, 506 people (364 males and 142 females; median age, 43 years) agreed to participate in the study. However, five participants were excluded from the masticatory ability measurement, as they were undergoing dental treatment and had temporary prostheses or restorations, leaving 501 participants in the study.

### Survey content

This survey consisted of a questionnaire and measurements. Variables were selected with reference to previous studies, and the following items were assessed [6, 7, 11, 12]: sex, age, body mass index (BMI), educational level, smoking, alcohol drinking, physical activity, oral health, and LS items. Physical activity was assessed using the University of California Los Angeles (UCLA) activity score [15], a simple scale ranging from 1 to 10. Participants reported their most appropriate activity level, with 1 defined as ‘no physical activity, dependent on others’ and 10 defined as ‘regular participation in impact sports’.

Sex, age, educational level (higher education, secondary education), smoking (none, past smoker, current smoker), alcohol drinking (none, a few times/month, a few times/week), and UCLA activity score (≥ 5 points or ≤ 4 points) were examined using a questionnaire. BMI was calculated using the measurements of height and weight on scales as weight (kg)/height (m)^2^.

### Dental status

In 2018, Tanaka et al. surveyed older adults aged 65 years and older and found that 1) fewer than 19 teeth, 2) low masticatory ability, 3) low articulation oral motor skills, 4) low tongue pressure, 5) difficulty eating tough foods, and 6) difficulty swallowing tea and soup corresponded to pre-oral frailty (one or two items) or oral frailty (three or more items) [16]. The participants in the present study were of working age and had greater compensatory abilities than the elderly population, and it was believed that those with multiple signs of oral frailty might go undetected. Therefore, two signs of oral frailty were investigated in the present study: the number of remaining teeth and masticatory ability. The JDA places the decrease in the number of remaining teeth in the early stage of oral frailty and the decrease in masticatory ability in the advanced stage because the decrease in interest in oral health leads to poor oral health care and increases the risk of losing teeth due to caries and periodontal disease [2]. In addition, a questionnaire established whether people received regular dental check-ups, as this is related to the decline in oral health literacy, which the JDA includes in the early stage of oral frailty [2].

The presence or absence of regular dental check-ups and the number of remaining teeth were surveyed using a questionnaire. For the number of remaining teeth, the investigator visually assessed the participants' oral cavities to control for information bias. Regarding the number of remaining teeth, the proportion of individuals aged 20–59 years with 19 or fewer teeth was expected to be approximately 5% of the Japanese average [17]. In this study, the number of remaining teeth was categorised into two groups based on the definition of oral frailty, ≥ 20 teeth and ≤ 19 teeth, and their association with LS was examined. This cut-off value was established in a survey of older people, and there is no previous cut-off value for working-age people. In other words, it is possible to identify high-risk groups for LS in the working-age population. Therefore, this study used this cut-off value for the number of remaining teeth to identify high-risk groups.

Masticatory performance was measured using colour-changing chewing gum (Masticatory Performance Evaluating Gum XYLITOL, Lotte Corporation, Tokyo, Japan). The colour-changing chewing gum (hereinafter referred to as ‘chewing gum’) changes from green to red when chewed well and mixed with saliva and is, therefore, a method for evaluating grinding and mixing ability [18, 19]. The measurement was carried out according to the manufacturer’s instructions [20]. Participants were instructed to chew the gum 60 times at a rate of once per second. The chewing gum was then pressed to a thickness of 1.5 mm. L*a*b* values were measured at five locations on the chewing gum using a colorimeter (Color Reader CR-20; Konica Minolta, Tokyo, Japan) based on the L*a*b* colour space model established by the International Commission on Illumination. The averages of the L*a*b* values were entered into the following equation to calculate masticatory ability: $\Delta E=\sqrt{{(L*-72.3)}^{2}+{(a*+14.9)}^{2}+{(b*-33.0)}^{2}}$.

In the present study, to identify the high-risk group for masticatory ability, we used the reference value ΔE = 37.2 and standard deviation of 6.1 given in the chewing gum manual and used the reference value of -1 standard deviation as the cut-off value (ΔE = 31.1) [20]. Participants were then classified into two groups (high and low).

### The LS risk test

LS risk was evaluated using a three-part test as follows.

### Stand-up test

This test evaluates leg strength by standing up from a specified seat height (40, 30, 20, or 10 cm) with one or both legs [3, 21]. Four seats of different heights were prepared, and the participants stood up from an easy to difficult height in the following order: 40, 30, 20, and 10 cm with both legs and 40, 30, 20, and 10 cm with one leg. While standing up, the patient was diagnosed as having achieved the height if he or she could maintain that posture for 3 s. Scores were assigned as 0, 1, 2, 3, 4, 5, 6, 7, and 8. A score of 0 is assigned if the participant is unable to stand up from a seat height of 40 cm with both legs, and a score of 8 is assigned if the participant is able to stand up from a seat height of 10 cm with one leg. Hence, the higher the score, the greater the participant’s ability. This test evaluates vertical mobility and reflects knee extension strength, primarily in the quadriceps muscles. Furthermore, sufficient joint range of motion, flexibility, and balance are required to stand up on one leg.

### Two-step test

This test measures stride length to evaluate walking ability [3, 22]. The procedure is as follows. (1) The participant stands at a starting line with both toes behind it. (2) The participant is instructed to walk two steps (as long as possible) and to keep both feet together. (3) The length of the two steps from the starting line to the tip of the toes where the participant stopped is measured. The score for the two-step test is calculated as follows: two steps (cm) ÷ height (cm). This test evaluates horizontal mobility, and test results correlate positively with the maximum walking speed. Walking also requires muscle strength, balance, and flexibility of the lower extremities.

### 25-question Geriatric Locomotive Function Scale

The 25-question Geriatric Locomotive Function Scale (GLFS-25) is a test that was developed by Seichi et al. [3, 23]. It is a self-administered, comprehensive scale consisting of 25 items: 4 questions on pain in the last month, 16 questions on activities of daily living in the last month, 3 questions on social functioning, and 2 questions on mental status in the last month. These 25 items are rated on a 5-point scale from no disability (0 points) to severe disability (4 points) and are added arithmetically to arrive at a total score (minimum = 0 points, maximum = 100 points). Thus, the higher the score, the more impaired the participant’s motor function.

### LS stage classification

On the basis of the results of the three measurements, the participants are classified as no LS, LS stage 1, LS stage 2, or LS stage 3 according to the following criteria [24].

LS stage 1: Difficulty standing up from a seat height of 40 cm with one leg during the stand-up test, or a two-step score of < 1.3 or GLFS-25 score of ≥ 7.

LS stage 2: Difficulty standing up from a seat height of 20 cm with both legs during the stand-up test, or a two-step score of < 1.1 or GLFS-25 score of ≥ 16.

LS stage 3: Difficulty standing up from a seat height of 30 cm with both legs during the stand-up test, or a two-step score of < 0.9 or GLFS-25 score of ≥ 24.

In the present study, to clarify the relationship between oral frailty and each of the three measurements, we categorised the participants into two groups according to the LS classifications of no LS or LS (LS stage 1, LS stage 2, and LS stage 3).

### Data analysis

The study population included approximately 200,000 people [25], as calculated from the number of employees in the included manufacturing companies. The sample size was set at 400, with a margin of error of ± 5%, confidence level of 95%, and response rate of 50%.

Fisher's exact test was used to examine the relationship between the presence of LS and two groups of remaining teeth and the presence of LS and two groups of masticatory ability, considering the small sample size case. In addition, logistic regression analysis was used to evaluate the relationship between the risk of LS according to the stand-up test result and the number of remaining teeth, and odds ratios and 95% confidence intervals were calculated. Potential confounders included sex, age, BMI, educational level, smoking, alcohol drinking, and UCLA activity score. Statistical analysis was performed using IBM SPSS Statistics, version 24 (IBM Japan, Inc., Chuo-ku, Tokyo, Japan), with a significance level of *p* < 0.05.

## Results

### Participants’ characteristics

Among the 501 participants in the masticatory ability measurement, 495 (354 males and 141 females, median age 43 years) with no missing data were analysed. The participants’ characteristics are summarised in Table 1. The median BMI values were 23.4 and 21.5 kg/m^2^, proportions of secondary education level were 40.9% and 61.0%, those of current smokers were 30.8% and 5.7%, and those of alcohol drinkers were 71.8% and 42.6% for males and females, respectively. The median UCLA activity score was 4.0 for both sexes. The proportions of participants who performed low physical activity were 52.1% and 78.0% for males and females, respectively. Less than half of the participants underwent regular dental check-ups. The median number of remaining teeth was 28 for males and females. The ΔE values were 39.9 for males and 37.7 for females. The groups are presented based on the number of remaining teeth and ΔE. Two percent of each sex group had ≤ 19 remaining teeth. Overall, 6.3% of participants were classified into the low masticatory ability group (4.0% of males, and 12.1% of females) on the basis of ΔE values. The prevalences of LS according to each measure were 13.3% for males and 35.5% for females in the stand-up test; 2.3% for males and 2.8% for females in the two-step test; and 17.5% for males and 24.1% for females in the GLFS-25. LS prevalence accounted for most of stage 1 cases for all measures.

**Table 1:** Distribution of participant characteristics by sex

	**Male**	**Female**
	***n* = 354**	***n* = 141**
Age (years)	43 (32, 49)	44 (32, 49)
≤ 29	65 (18.4%)	26 (18.4%)
30–39	87 (24.6%)	25 (17.7%)
40–49	118 (33.3%)	58 (41.1%)
50–59	64 (18.1%)	29 (20.6%)
60–65	20 (5.6%)	3 (2.1%)
BMI	23.4 (21.9, 25.6)	21.5 (20.1, 24.7)
Educational level		
Secondary education	144 (40.9%)	86 (61.0%)
Higher education	208 (59.1%)	55 (39.0%)
Smoking		
None	204 (57.6%)	131 (92.9%)
Past smoker	41 (11.6%)	2 (1.4%)
Current smoker	109 (30.8%)	8 (5.7%)
Alcohol drinking		
None	100 (28.2%)	81 (57.4%)
A few times/month	66 (18.6%)	28 (19.9%)
A few times/week	188 (53.1%)	32 (22.7%)
UCLA activity score	4 (4, 9)	4 (4, 4)
≤ 4 points	184 (52.1%)	110 (78.0%)
≥ 5 points	169 (47.9%)	31 (22.0%)
Number of remaining teeth	28 (27, 28)	28 (27, 28)
≤ 19	7 (2.0%)	3 (2.1%)
Masticatory ability (ΔE)	39.9 ± 4.8	37.7 ± 6.5
Low masticatory ability	14 (4.0%)	17 (12.1%)
Dental check-ups		
Not receiving	209 (59.0%)	73 (51.8%)
Prevalence of LS		
Stand-up test	47 (13.3%)	50 (35.5%)
LS stage 1	46 (13.0%)	49 (34.8%)
LS stage 2	0 (0%)	1 (0.7%)
LS stage 3	1 (0.3%)	0 (0%)
Two-step test	8 (2.3%)	4 (2.8%)
LS stage 1	7 (2.0%)	4 (2.8%)
LS stage 2	1 (0.3%)	0 (0%)
LS stage 3	0 (0%)	0 (0%)
GLFS-25	62 (17.5%)	34 (24.1%)
LS stage 1	44 (12.4%)	28 (19.9%)
LS stage 2	12 (3.4%)	2 (1.4%)
LS stage 3	6 (1.7%)	4 (2.8%)

Values are presented as *n* (%), median (lower quartile, upper quartile), or mean ± standard deviation

Low masticatory ability was classified according to the cut-off value, ΔE = 31.1

LS: Stand-up test score < 5, difficulty standing on one foot (either leg) from a seat height of 40 cm; two-step test score < 1.3; GLFS-25 score ≥ 7

*Abbreviations*: *BMI* Body mass index, *UCLA* University of California Los Angeles, *LS* Locomotive syndrome, *GLFS-25* 25-question Geriatric Locomotive Function Scale

### Relationship between oral frailty and LS

The relationship between the number of remaining teeth and LS is shown in Table 2. In the stand-up test, a significant relationship between the number of remaining teeth and LS was observed, with a higher percentage of those with ≤ 19 remaining teeth being in the LS group (*p* < 0.05). No relationship between the two masticatory ability groups and LS could be confirmed (*p* > 0.05; Table 3).

**Table 2:** Relationship between the number of remaining teeth and LS

	**Stand-up test**		***p*- value**	**Two-step test**		***p*-value**	**GLFS-25**		***p*-value**
	**No LS**	**LS**		**No LS**	**LS**		**No LS**	**LS**	
Number of remaining teeth									
≥ 20 (*n* = 485)	393 (98.7%)	92 (94.8%)	0.029^*^	473 (97.9%)	12 (100%)	> 0.999	393 (98.5%)	92 (95.8%)	0.108
≤ 19 (*n* = 10)	5 (1.3%)	5 (5.2%)		10 (2.1%)	0 (0%)		6 (1.5%)	4 (4.2%)	

Values are presented as *n* (%)

LS: Stand-up test score < 5, difficulty standing on one foot (either leg) from a seat height of 40 cm; two-step test score < 1.3; GLFS-25 score ≥ 7

*Abbreviations*: *LS* Locomotive syndrome, *GLFS-25* 25-question Geriatric Locomotive Function Scale

^*∗*^*p* < 0.05 by Fisher's exact test

**Table 3:** Relationship between masticatory ability and LS

	**Stand-up test**		***p*-value**	**Two-step test**		***p*-value**	**GLFS-25**		***p*-value**
	**No LS**	**LS**		**No LS**	**LS**		**No LS**	**LS**	
Masticatory ability									
High (*n* = 464)	373 (93.7%)	91 (93.8%)	> 0.999	454 (94.0%)	10 (83.3%)	0.169	376 (94.2%)	88 (91.7%)	0.351
Low (*n* = 31)	25 (6.3%)	6 (6.2%)		29 (6.0%)	2 (16.7%)		23 (5.8%)	8 (8.3%)	

Values are presented as *n* (%)

LS: Stand-up test score < 5, difficulty standing on one foot (either leg) from a seat height of 40 cm; two-step test score < 1.3; GLFS-25 score ≥ 7

*Abbreviations*: *LS* Locomotive syndrome, *GLFS-25* 25-question Geriatric Locomotive Function Scale

In addition, logistic regression analysis based on the presence of LS in the stand-up test showed odds ratios of 5.99, 4.38, 0.96, 1.14, and 2.30 for the group with ≤ 19 remaining teeth, female sex, age, BMI, and UCLA activity score of ≤ 4 points in participants with LS, respectively (Table 4).

**Table 4:** Results of logistic regression analysis of the relationship between the stand-up test result (No LS or LS) and factors

Variable	*p*-value	OR	95% CI	
			**Lower**	**Upper**
Sex				
Male	< 0.001^*^	ref	2.42	7.92
Female		4.38		
Age (years)	< 0.001^*^	0.96	0.94	0.98
BMI (kg/m^2^)	< 0.001^*^	1.14	1.06	1.23
Educational background				
Higher education	0.577	ref	0.52	1.45
Secondary education		0.86		
Smoking				
None	0.656	ref		
Past smoker	0.423	1.46	0.58	3.70
Current smoker	0.519	1.24	0.64	2.41
Alcohol drinking				
None	0.716	ref		
A few times/month	0.897	0.96	0.49	1.87
A few times/week	0.427	0.79	0.44	1.41
UCLA activity score				
≥ 5 points	0.004^*^	ref	1.31	4.05
≤ 4 points		2.30		
Number of remaining teeth				
≥ 20	0.014^*^	ref	1.44	24.95
≤ 19		5.99		

Covariates: sex, age (years), BMI (kg/m^2^), educational level, smoking, alcohol drinking, UCLA activity score, and number of remaining teeth

*Abbreviations*: *LS* Locomotor syndrome, *BMI* Body mass index, *UCLA* University of California Los Angeles, *OR* Odds ratio, *CI* Confidence interval, *ref* reference

^*^p < 0.05, Hosmer–Lemeshow test: *p* = 0.560

## Discussion

In the present study, we investigated the relationship of the number of remaining teeth and masticatory ability with LS in the working-age population. The results showed that according to the stand-up test result, the group with ≤ 19 remaining teeth had a higher rate of LS than the group with ≥ 20 remaining teeth, with an odds ratio of 5.99.

The participants had a median age of 43 years. The age range of the participants was 20–49 years, with 80% of the population being 20–49 years and a small percentage being 50 years or older. The BMI was in the normal range for both males and females.

Regarding the number of remaining teeth, as of 2019, the average percentage of Japanese aged 20–59 years with 19 or fewer teeth was approximately 5%, and the number of teeth decreased with age [17]. The results of the present study showed similar results; the number of remaining teeth of 19 or fewer was 2%. Masticatory ability was comparable to the reference values for males and females. Approximately 6% of participants were classified in the group with low masticatory ability. Overall, those with signs of oral frailty were rare. It is assumed that this was a reasonable result since the target population was the working-age population with an average age of 43 years. However, the lack of regular dental check-ups is a cause for concern, with only approximately 40% of people having regular dental check-ups. Japanese statistics showed that the rate of consultation among the working-age population is lower than that of the elderly population, with the rate of consultation among those 49 years of age and younger being less than 50% [26]. Therefore, the goal is to have 60% of the population receiving annual dental check-ups. We believe this relates to the dental healthcare system in the Japanese workplace. All workers in Japan are required to undergo a medical check-up by a physician but are not obligated to undergo dental examinations, except for those engaged in special work involving the handling of acids. Thereby, outside of working hours, the working-age population must voluntarily engage in self-care through dental check-ups and dental visits conducted by the local government that has jurisdiction over their area of residence for early detection and early treatment of tooth decay and periodontal disease. However, it has been noted that many people find it difficult to do so due to their busy work schedules and time constraints [27]. For this reason, half of the study participants were also considered unable to attend regular dental appointments.

As mentioned earlier, regular dental check-ups are fundamental and very important to prevent oral frailty since poor oral health literacy is considered in the early stage of oral frailty and increases the risk of tooth loss due to tooth decay and periodontal disease [2]. The working-age population's access to dental health services needs to be improved.

As mentioned earlier, in working-age males and females, it was reported that LS stage 1 was present in 21.7% and 25.0% of those younger than 40 years of age, 31.6% and 41.1% of those aged 40–49 years, and 41.5% and 42.1% of those aged 50–59 years, respectively [5]. This indicates a high incidence of LS stage 1 in the working-age population, approximately over 30%. The prevalence of LS based on the criteria of the two-step test was approximately 1% [11, 12], with a very low incidence in the working-age population, which is consistent with the results of the present study. The prevalences of LS based on GLFS-25 criteria were reported to be 17% and 25% for males and females, respectively, by Otsuki et al. and 15.2% and 22.8% for males and females, respectively, by Nishimura et al., which are in agreement with the results of this study [11, 12]. Additionally, Akahane et al. reported a prevalence of LS of 5.3%; the LS cut-off value they used was ≥ 16 points, corresponding to LS stages 2 and 3 in this study, and the results were consistent with ours [7]. The prevalences of LS based on the criteria of the stand-up test have been reported to be 6.5% and 12.6% [11] and 3.1% and 8.8% [12] in males and females, respectively. The results of the present study showed that the prevalence of LS in males was in line with those of previous studies, whereas the prevalence in females was very high at 35%. LS is more prevalent in females compared to males [28]. In the present study, females were also 4.8 times more likely than males to have the condition. Ogata et al. found that from the age of 20 to 49 years, females scored significantly worse than males in the stand-up test [29]. Narumi et al. found that knee extension muscle strength, as assessed by the stand-up test, was significantly lower in females than in males in each age group from 20 to 89 years [30].

The results of the present study further supported the findings of previous studies. The female participants in this study were characterised by the fact that approximately 60% of the participants were aged 40–59 years. Yoshimura et al. reported that more than 40% of women aged 40–59 had LS [5]. Another characteristic of the females was that 80% them had low physical activity levels, with a UCLA score of ≤ 4 points or less. Those with low physical activity were 2.3 times more likely to have LS than those with high physical activity; those with low physical activity (UCLA score of ≤ 4 points) had a higher prevalence of LS, and Ohtsuki et al. reported that approximately 80% of those with LS had a UCLA score of ≤ 4 points [11, 12]. These factors were predicted to affect the high prevalence of LS among females in the present study.

Regarding the effect of age, the odds ratio was slightly lower, at 0.96 for age, in contrast to results of previous studies [11–14]. This could be because more younger participants (20–30 years) had worse scores.

In the stand-up test, the group with ≤ 19 remaining teeth had a higher LS rate than the group with ≥ 20 remaining teeth, and the results of the logistic regression analysis showed that the group with ≤ 19 remaining teeth had a 5.99-times higher LS onset. In the stand-up test, participants with LS may not have sufficient knee extensor strength to stand on one leg from a seat height of 40 cm. Knee extensor strength is significantly increased by clenching through a phenomenon called simultaneous activation enhancement [31, 32]. Clenching also increases muscle activity in the sternocleidomastoid and trapezius muscles, which are involved in maintaining head position when standing [33]. It also stimulates the H-reflex in the tibialis anterior and soleus muscles, which aids postural control associated with the forward and upward shift of the centre of gravity during standing [34–36]. In areas of tooth loss, maximum occlusal force is reduced [37, 38]. In other words, it has been speculated that reduced clenching force may have resulted in a lack of afferent sensory input from periodontal ligament receptors [39], affecting knee extensor strength and postural control. The effects of clenching during the standing test should be investigated in the future.

Previously, Akahane et al. and Tominaga et al. reported significant associations between GLFS-25 scores and the number of remaining teeth in populations with mean ages of 58 and 69.9 years, respectively [7, 9]. Nagilla et al. reported a significant association between Loco-Check LS scores and the number of remaining teeth in a population with a mean age of 59 years [8]. The participants in the previous study were older than those in the present study, and the previous study used a rating index derived from the participants' subjectivity. The present study found a significant association between the number of remaining teeth and presence of LS according to the stand-up test, a dynamic and objective assessment measure. To the best of our knowledge, this is the first study to examine the relationship between the presence of LS according to the stand-up test and the number of remaining teeth in a working-age population.

As mentioned earlier, LS stage 1 occurs before physical frailty, whereas LS stage 3 is almost identical to physical frailty. Tanaka et al. reported a significant increase in physical frailty two years later in those with one or two signs of oral frailty compared with those with no signs of oral frailty [16]. Our study identified those in the pre-stage of physical weakness based on the stand-up test result for LS and found that standing movements were associated with the number of remaining teeth, supporting Tanaka et al.'s findings [16]. In conjunction with the LS test assessment, the number of remaining teeth may be an effective tool for identifying those at risk for physical frailty at an earlier stage. The purpose of LS examinations in our working-age population was to prevent occupational accidents such as falls at work. Nishimura et al. stated that to prevent LS, it is necessary to develop exercise habits early and create an environment where the working-age population can exercise at work [40]. A decrease in the number of remaining teeth was associated with difficulty in standing movements in the present study, which suggests that oral frailty prevention measures should be used to prevent LS in the working-age population.

This study has some limitations. First, this study was limited to four regional companies, and the number of participants was limited; additionally, as workplace surveys tend to include generally healthy people, there may have been a selection bias. Therefore, it is important to remember that the results reflect the workplace setting, necessitating the expansion to geographical areas to generalise the study’s findings. Secondly, as the target population was of working age, there were fewer people with < 19 remaining teeth. Thus, this association should be interpreted with caution. Larger studies are needed to estimate the effect more precisely. Thirdly, the number of remaining teeth and masticatory ability were assessed, but bite and clenching were not, so a more detailed assessment of occlusion is needed to confirm the relationship with LS. Finally, because this is a cross-sectional study, the causal relationship between the decrease in the number of remaining teeth and LS is unclear. Longitudinal studies are needed to clarify these causal relationships.

## Conclusion

The present study investigated the relationship between oral frailty and LS in a working-age population. Signs of oral frailty, such as tooth loss and masticatory ability, were identified, and a higher proportion of LS was found in the group with ≤ 19 remaining teeth.

## Data Availability

The datasets used and/or analysed during the current study are available from the corresponding author upon reasonable request.

## Abbreviations

LS: Locomotive syndrome
BMI: Body mass index
GLFS-25: 25-Question Geriatric Locomotive Function Scale
OR: Odds ratio
CI: Confidence interval
JDA: Japanese Dental Association
JOA: Japanese Orthopaedic Association
UCLA: University of California Los Angeles
